# Progressive Impairment of Mismatch Negativity Is Reflective of Underlying Pathophysiological Changes in Patients With First-Episode Psychosis

**DOI:** 10.3389/fpsyt.2020.00587

**Published:** 2020-06-18

**Authors:** Silvia Kyungjin Lho, Minah Kim, Jihye Park, Wu Jeong Hwang, Sun-Young Moon, Sanghoon Oh, Jun Soo Kwon

**Affiliations:** ^1^Department of Psychiatry, Seoul National University College of Medicine, Seoul, South Korea; ^2^Department of Neuropsychiatry, Seoul National University Hospital, Seoul, South Korea; ^3^Department of Brain and Cognitive Sciences, Seoul National University College of Natural Sciences, Seoul, South Korea; ^4^Institute of Human Behavioral Medicine, SNU-MRC, Seoul, South Korea

**Keywords:** first-episode psychosis, mismatch negativity, event-related potential, longitudinal change, pathophysiology

## Abstract

**Background:**

Although mismatch negativity (MMN) is associated with the pathophysiology of schizophrenia, whether MMN progressively worsens during the initial years of psychotic disorder has not yet been sufficiently studied. We aimed to investigate whether longitudinal reduction of MMN occurs in patients with first-episode psychosis (FEP) and whether it is reflective of change in cognitive functioning or clinical status.

**Methods:**

MMN and the clinical status of 25 patients with FEP were measured and the Trail Making Test (TMT) was administered at baseline and reassessed after 1 year of usual treatment. The MMN of 25 matched healthy controls (HCs) was measured at baseline. Repeated-measures analysis of variance was used to compare MMNs at baseline among the groups, and paired t-test was utilized to compare the baseline and 1-year MMN amplitudes of FEP patients. To identify the association between changes in MMN and changes in cognitive, symptomatic, or functional status over 1 year, multiple regression analysis was used to control for other possible confounders.

**Results:**

MMN amplitudes at baseline were significantly attenuated in FEP patients compared to those in HC. The 1-year follow-up MMN amplitude decreased significantly at the Fz electrode site in the FEP group. Additionally, the decreased MMN amplitude significantly correlated with worsened TMT part B (TMT-B) performance over 1 year but did not correlate with symptomatic or functional improvement.

**Conclusions:**

FEP patients with an MMN amplitude reduction showed worsening of cognitive functioning, which might reflect pathophysiological progression during the early years of a psychotic episode.

## Introduction

Schizophrenia has historically been considered a progressive and deteriorating psychotic illness characterized by disruption of mental functions (1–3), which was implied by Kraepelin’s concept of “dementia praecox” (4). Kraepelin emphasized the progressive nature of the illness, which he thought might be explained by neurobiological changes (5). Until now, neuroimaging research regarding patients with schizophrenia has shown decreased gray matter volumes and increased ventricular size associated with cognitive impairment, suggesting that structural alterations appear during the course of the illness (6–9).

However, it is yet unclear when and how brain alterations arise over the course of schizophrenia. Especially in first-episode psychosis (FEP) patients, there are inconsistent reports regarding whether and when progressive brain changes occur. Several studies have reported that structural and functional brain changes occur during the first episode of schizophrenia, especially in the initial years (10–12). Additionally, impairment of cognitive function occurs and persists regardless of amelioration of psychotic symptoms (13, 14), indicating that underlying neurobiological changes occur during the early stages of the illness. On the other hand, some studies have not seen such alterations in the brain in FEP (15, 16). One possible explanation for the conflicting results is that the clinical outcomes of schizophrenia vary among patients, implying the presence of heterogeneous disease course for schizophrenia (17). Another possible explanation is that previous studies have mostly been structural studies that might be less sensitive to subtle pathophysiological changes (18). Therefore, it would be helpful to study electrophysiological markers that are closely related to cognitive function and sensitive to change with high temporal resolution (19).

Auditory mismatch negativity (MMN) is a component of event-related potential that can be elicited when a sequence of repetitive sounds is interrupted by a deviant oddball sound that differs in its physical properties such as frequency, duration or intensity. MMN is considered to reflect an automatic detection process of differentiating a standard frequent sound from a deviant oddball sound at the preconscious level (20). MMN has been demonstrated to represent *N*-methyl-d-aspartate glutamate receptor function (21) and is known to be associated with schizophrenia pathophysiology. MMN impairment is reliably reported in patients with schizophrenia spectrum disorder, including during the early stages of psychosis and chronic schizophrenia (22–24). The consistent findings of MMN impairments showing correlation with poor functional status in chronic schizophrenia (25–27) and MMN representing a predictive marker for conversion to psychosis or remission in clinical-high risk (CHR) populations (28–30) indicate the clinical importance of MMN as a biomarker in schizophrenia. In addition, MMN deficits have been associated with diminished higher-order cognitive functions, including working memory and executive function, in schizophrenia patients (27, 31, 32). Considering that MMN is a component related to functional outcome or prognosis at each stage of the disease and is also associated with diminished cognitive functions that may reflect pathophysiological changes in the brain, MMN can be a promising marker for investigating pathophysiological changes during the disease course of schizophrenia.

Previous cross-sectional studies indicated that MMN impairment may progress along the course of the illness because the impairment is more severe in the chronic stage than in the relatively early stage, such as recent-onset or first-episode psychosis (FEP) (23). Additionally, the MMN in CHR patients was impaired compared to that in healthy subjects but relatively less impaired than in patients who developed schizophrenia (24, 33). A recent meta-analysis reported that MMN impairment appeared to worsen over time since the effect size increased (CHR [effect size = 0.40] < FEP [0.42] < chronic schizophrenia [0.81]) along with illness progression (24). However, the duration of illness showed no correlation with the MMN effect size. The author explained the result by the possibility of nonlinear deterioration of the MMN, suggesting that MMN impairment gradually progresses during the first year or two after onset, and this progression will stabilize after the critical period (24).

To clarify the natural course of MMN, prospective studies are needed to reveal the longitudinal course of MMN and related clinical status, especially in the early stages of schizophrenia. To our knowledge, there have been four longitudinal studies regarding MMN in FEP so far, but the results were inconsistent across the studies. Kaur et al. (34) showed that duration deviant MMN (dMMN), namely, MMN in response to duration deviant tones, progressively became impaired. Additionally, Devrim-Ucok et al. (35) and Salisbury et al. (36) reported that frequency deviant MMN (fMMN), namely, MMN in response to frequency deviant tones, worsened over time in FEP. However, a study published in 2017 that longitudinally followed up both dMMN and fMMN did not show progression of MMN impairment (37). These studies were insufficient to clarify the nature of MMN change since Kaur et al. (34) included patients with disorders other than psychotic disorders such as bipolar spectrum disorder, and the duration of follow-up varied among participants in two studies (35, 37). None of the previous studies found a significant association of MMN change with changes in cognitive functioning or clinical status.

Thus, in this study, we aimed to examine whether progressive impairment of MMN occurs during the early years of psychotic disorder by repeated measurements of MMN over 1 year in patients with FEP. In addition, the association of MMN change with changes in cognitive functioning as well as symptomatic or functional improvement was investigated for 1 year to reveal whether MMN change is reflective of underlying pathophysiological change or clinical improvements. We hypothesized that MMN would show progressive worsening over 1 year in FEP patients and be associated with worsening cognitive functioning, which may be reflective of the pathophysiological progression of the psychotic disorder during the early course of schizophrenia.

## Materials and Methods

### Participants

We recruited 36 patients with FEP between April 2017 and June 2018 *via* the inpatient and outpatient clinics in the Department of Neuropsychiatry of Seoul National University Hospital (SNUH) and the Seoul Youth Clinic (www.youthclinic.org) (38). Among these patients, 25 patients with FEP participated in both baseline and 1-year MMN measurements. The other 11 patients with FEP declined to participate in the follow-up MMN measurements. A patient with FEP was defined as an individual who met the Diagnostic and Statistical Manual of Mental Disorders, Fourth Edition (DSM-IV) criteria of schizophrenia, schizoaffective disorder, or schizophreniform disorder, and the onset was within the last two years to include patients who were in the first or second year of psychosis (39). FEP patients had their diagnosis confirmed with a clinical interview by certified psychiatrists using the Structured Interview for the Diagnostic and Statistical Manual of Mental Disorders, Fourth Edition Axis I Disorders (SCID-1). HCs were recruited through Internet advertisements and excluded subjects with a past or current DSM-IV Axis I psychiatric history or family history of schizophrenia within third-degree relatives. Additionally, it was confirmed that no participants had a history of substance abuse or dependence (except nicotine), severe head trauma, neurological disease, severe medical illness, sensory impairments, or intellectual disability (intelligence quotient [IQ] < 70) for both groups.

All study participants understood the study procedures and provided written informed consent according to the Declaration of Helsinki. This study protocol was approved by the Institutional Review Board of SNUH.

### Demographic Factors, Clinical Characteristics, and Medication Usage

The demographic factors included age, sex, and years of education. IQ measurement and handedness were assessed using the Korean Wechsler Adult Intelligence Scale (40) and the Annett Handedness Inventory (41), respectively. The evaluations of duration of untreated psychosis (DUP) and duration of illness (DOI) were based on the medical records of the patients and interviews with the patients and their family members. Clinical statuses of FEP patients were measured at baseline and reassessed after a 1-year treatment using the Positive and Negative Syndrome Scale (PANSS) and Global Assessment of Functioning (GAF). Several neurocognitive functions including processing speed, working memory, task-switching ability, and mental flexibility were measured by the Trail Making Test (TMT) at baseline and after 1 year in patients with FEP. Processing speed was assessed using the TMT part A (TMT-A). Working memory and task-switching abilities were measured by the TMT part B (TMT-B). The ratio score of the TMT-B to TMT-A (TMT B/A) was assessed to minimize visuospatial and working memory demands, thus specifically measuring mental flexibility (42). Calculations of the changes in the symptoms, functional status, and neurocognitive functions were performed by subtracting the 1-year follow-up scores of the PANSS, GAF, and TMT-A and TMT-B from the scores measured at baseline. The usage of antipsychotics was documented and converted into a daily olanzapine equivalent dose (43).

### ERP Measurement

The recording protocol of the electroencephalography (EEG) utilized in this study was identical to that previously used in our laboratory (33). While subjects concentrated on a “Where’s Wally?” picture book, the auditory stimuli (80 dB; 10 ms rise/fall time) were presented binaurally *via* a STIM2 sound generator (Neuroscan, El Paso, TX, USA) with a fixed intertrial interval of 600 ms. The participants were presented with a pseudorandom series of two types of stimuli, which differed in duration, composed of standard/frequent stimuli (50 ms tones, 81.8% (982 of 1,200 trials)) and target/infrequent stimuli (100 ms tones, 18.2% (218 of 1,200 trials)).

EEGs were continuously recorded by a Neuroscan SynAmps 128-channel system using the quick-cap with 64 electrodes placed according to the international 10–20 system (Neuroscan, El Paso, TX, USA). The left and right mastoids were used as the reference. The EEG signals were digitized at a sampling rate of 1 kHz and filtered from 0.05 to 100 Hz online. Electrodes placed below and on the outer canthus of the left eye were used to obtain horizontal and vertical electrooculograms (EOGs). The impedance of all electrodes was less than 5 kΩ.

We used Curry 7 software (Compumedics, Charlotte, NC, USA) to analyze continuous EEG data. Bad channels, which contained persistent artifacts throughout the trials, were reconstructed using a linear interpolation of the surrounding channels (up to 7% per participant). The removal of eye movement artifacts was performed by utilizing an artifact reduction algorithm in Curry 7 software (44). The EEG data were re-referenced to a common average reference and filtered with a bandpass of 0.1 to 30 Hz. The data were subsequently epoched between 100 ms prestimulus and 400 ms poststimulus and baseline corrected using the mean amplitude during 100 ms prior to stimulus onset. Epochs that contained EEG voltages exceeding ±75 μV were automatically rejected as artifacts, and the number of remaining epochs did not differ between the patients with FEP and the HCs (independent t-test; *t* = −0.162, *p* = 0.872, number of remaining epochs: 183.9 ± 32.2 vs. 182.4 ± 35.6, respectively). Additionally, the number did not differ between the patients with FEP at baseline and 1 year (paired t-test; *t* = 0.546, p = 0.590, number of remaining epochs: 183.9 ± 32.2 vs. 179.4 ± 32.8, respectively). MMN amplitude and latency were identified using a peak detection method that identifies the most negative deflection point between 130 and 250 ms poststimulus at Fz and FCz. Two electrodes were selected because MMN shows maximal amplitude in the frontocentral area (45).

### Statistical Analysis

Independent samples t-test (equal variances) or Welch’s t-test (unequal variances) for continuous variables and χ^2^ analysis or Fisher’s exact test for the categorical data were used to compare the demographic and clinical characteristics of the FEP patients and HC participants. To compare the differences in the MMN amplitudes and latencies, repeated-measures analysis of variance (ANOVA) was performed with the between-subject factor of group (FEP vs. HC) and the within-subject factor of electrode site (Fz and FCz). Furthermore, we compared the MMN amplitude and latency at each site *via* independent samples t-test to identify the specific electrodes that showed significant group differences for MMN. Group comparisons of MMN amplitudes and latency for FEP at baseline and at the 1-year follow-up were performed using paired t-tests. A multiple regression analysis with backward selection was used to identify significant factors that correlate with the changes in clinical outcomes such as symptoms (PANSS total, positive, negative, and general), general functional status (GAF), and cognitive functions (TMT-A, B, and B/A). The following factors, which were considered to be associated with the changes in clinical outcomes, were initially included in the analysis as independent variables: the MMN change at Fz (subtracting the 1-year MMN amplitude or latency from the baseline amplitude or latency); demographic factors, including age, sex, handedness, level of education (education years) and IQ; the duration of untreated psychosis (DUP); PANSS total, positive, negative, general subscale scores, GAF, TMT-A, B or B/A at baseline; and medication use during the 1-year prior to follow-up (mean daily olanzapine equivalent dose of antipsychotics). IBM SPSS version 23 (IBM) was used for statistical analyses. The threshold for significance was set at P < 0.05.

## Results

### Subject Characteristics

**Table 1** summarizes the clinical and demographic factors of the FEP patients at baseline and the HCs. No differences were identified for age, sex, handedness, IQ or years of education between the FEP patients and HCs. FEP patients at baseline and HCs showed differences in reaction time in TMT-A but not in TMT-B and TMT B/A. **Table 2** shows the clinical characteristics and MMN of patients with FEP at baseline and after 1 year of usual treatment. Scores on PANSS and GAF significantly improved after 1 year of usual treatment. However, there was no difference in reaction time of TMT-A, B, and B/A between baseline and 1-year follow-up. The olanzapine equivalent dose of antipsychotics showed no significant difference between FEP baseline and 1 year. There were no significant differences in demographic or clinical characteristics between the FEP follow-up group (N = 25) and follow-up loss group (N = 11) (**Supplementary Table 1**).

**Table 1:** Demographic characteristics, neurocognitive functioning, and mismatch negativity (MMN) of patients with first-episode psychosis (FEP) and healthy control (HC) subjects at baseline.

	FEP baseline (N = 25)		HC (N = 25)		Statistical Analysis^a^	
	Mean	SD	Mean	SD	χ^2^ or T	P
Age (years)	23.1	5.2	23.1	5.2	0.000	1.000
Sex (male/female)	10/15		10/15		0.000	1.000
Handedness (right/left)	13/2		15/0		–	0.490
IQ	101.9	16.3	112.9	12.5	0.695	0.108
Education (years)	13.6	2.3	14.1	2.0	0.835	0.409
DUP (months)	5.7	4.0	–	–	–	–
DOI (months)	9.1	6.9	–	–	–	–
TMT-A^b^	26.0	7.7	21.8	6.1	2.06	0.045^*^
TMT-B^b^	78.8	54.3	55.7	15.7	1.966	0.060
TMT B/A^b^	3.01	1.50	2.64	0.74	1.069	0.293
MMN amplitude (µV)						
Fz	−1.8	1.0	−2.7	1.0	−3.068	0.004^**^
FCz	−1.7	1.3	−2.8	1.1	−3.291	0.002^**^
MMN latency (ms)						
Fz	181.4	23.4	181.2	28.3	0.016	0.987
FCz	186.6	17.5	180.6	30.0	0.863	0.392

IQ, Intelligent quotient; DUP, Duration of untreated psychos; DOI, Duration of illness; TMT-A, Trail Making Test part A; TMT-B, Trail Making Test part B; TMT B/A, Ratio score of the Trail Making Test part B to part A; MMN, Mismatch negativity.

a Independent samples t-test for continuous variables and χ^2^ test for categorical variables.

b Missing values, n: TMT-A, B, and B/A of FEP baseline, 2.

*The mean difference is significant at the 0.05 level.

**The mean difference is significant at the 0.005 level.

**Table 2:** Clinical characteristics, neurocognitive functioning, and MMN of patients with first-episode psychosis (FEP) at baseline and 1 year follow-up.

	FEP baseline (N = 25)		FEP 1 year (N = 25)		Statistical Analysis^a^	
	Mean	SD	Mean	SD	χ^2^ or T	P
Follow-up duration (days)	–	–	364.6	58.5	–	–
PANSS						
Total	58.6	20.2	43.3	11.6	4.060	<0.001^**^
Positive symptoms	13.8	5.2	9.3	2.4	4.670	<0.001^**^
Negative symptoms	15.7	7.2	12.4	5.1	2.373	0.026^*^
General symptoms	29.0	10.0	22.1	5.6	3.671	0.001^**^
TMT-A^b^	26.0	7.7	27.7	6.4	−1.009	0.324
TMT-B^b^	78.8	54.3	63.2	20.4	0.972	0.342
TMT B/A^b^	2.88	1.39	2.34	0.57	1.069	0.109
GAF	54.5	17.7	63.6	13.4	−3.436	0.002^**^
Antipsychotic dose^c^	17.2	13.2	18.9	13.9	−0.964	0.345
MMN amplitude (µV)						
Fz	−1.8	1.0	−1.3	1.0	−2.129	0.044^*^
FCz	−1.7	1.3	−1.5	1.2	−0.999	0.328
MMN latency (ms)						
Fz	181.4	23.4	206.4	32.9	−3.281	0.003^**^
FCz	180.6	30.0	206.8	31.1	−3.199	0.004^**^

PANSS, Positive and Negative Syndrome Scale; TMT-A, Trail Making Test part A; TMT-B, Trail Making Test part B; TMT B/A, Ratio score of Trail Making Test part B to part A; GAF, Global Assessment of Functioning; MMN, mismatch negativity.

a Paired t-test for FEP baseline and FEP 1 year.

b Missing values, n: TMT-A, B and B/A of FEP baseline, 2; TMT-A of FEP 1 year, 2; TMT-B and B/A of FEP 1 year, 3.

c Mean olanzapine equivalent dose.

*The mean difference is significant at the 0.05 level.

**The mean difference is significant at the 0.005 level.

### MMN of FEP Baseline and HC Groups

**Figure 1:**
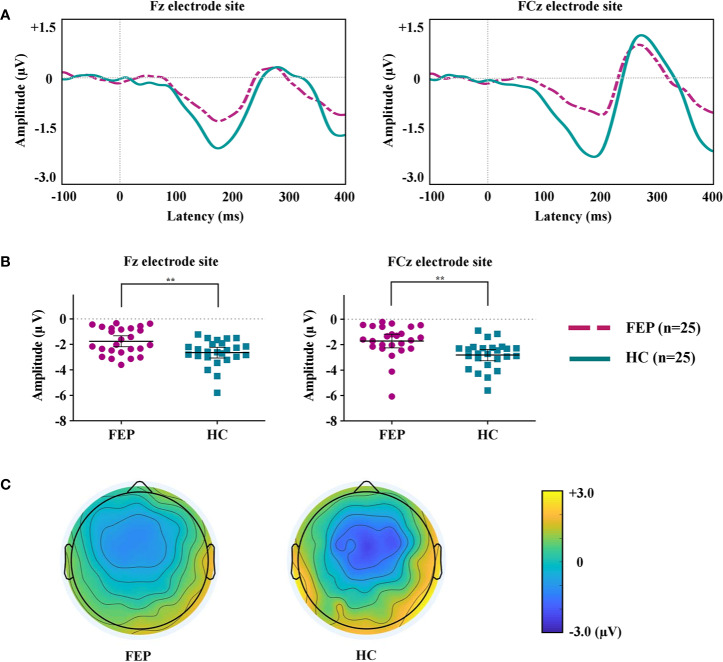
**(A)** Grand-averaged mismatch negativity (MMN) waveforms at the Fz and FCz electrode sites in patients with first-episode psychosis (FEP) and healthy control (HC) subjects. **(B)** The MMN amplitudes at the Fz and FCz electrode sites across the groups. The horizontal and vertical lines in the group indicate means with 95% confidence intervals (95% CIs) ^**^ indicate that the mean differences are significant at the 0.005 level. **(C)** Two-dimensional MMN topographic maps across FEP and HC groups.

**Figure 1A** shows the grand-averaged MMN waveforms. **Figure 1B** displays the MMN peak amplitudes at the Fz and FCz electrode sites across the FEP and HC groups. **Figure 1C** illustrates topographic maps of the MMN amplitudes of the FEP and HC participants. A repeated-measures ANOVA with the between-subject factor of group (FEP vs. HC) and within-subject factor of electrode sites (Fz and FCz) indicated a significant main effect of group (F = 11.766, *p* = 0.001), but no significant effects of electrode site (F = 0.320, *p* = 0.574) or group by electrode interaction (*F* = 0.826, *p* = 0.368) were present. The independent samples t-tests indicated that the MMN amplitudes at Fz (*p* = 0.004) and FCz (*p* = 0.002) were smaller in the FEP patients than in the HCs (**Table 1**). With respect to MMN latency at baseline, no significant effects of group (*F* = 0.264, *p* = 0.610), electrode site (*F* = 0.338, *p* = 0.564) or group by electrode interaction (*F* = 0.553, *p* = 0.461) were present. There was no difference in amplitudes or latencies of MMN between the FEP follow-up group (N = 25) and follow-up loss group (N = 11) (**Supplementary Table 2**).

### MMN of FEP Baseline and FEP 1 Year

**Figure 2:**
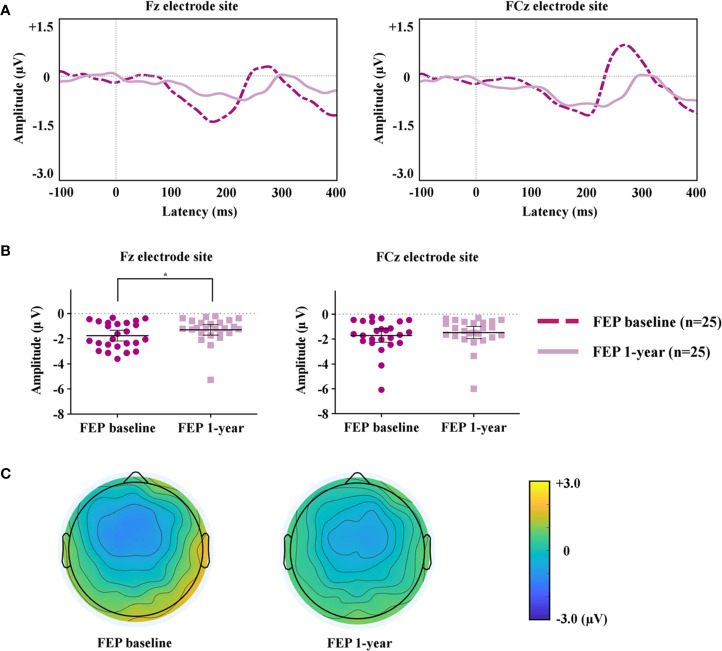
**(A)** Grand-averaged mismatch negativity (MMN) waveforms at the Fz and FCz electrode sites in patients with first-episode psychosis (FEP) baseline and FEP 1 year. **(B)** The MMN amplitudes at the Fz and FCz electrode sites at FEP baseline and 1 year. The horizontal and vertical lines in the group indicate means with 95% confidence intervals (95% CIs) ^*^ indicates that the mean differences are significant at the 0.05 level. **(C)** Two-dimensional MMN topographic maps across patients with FEP baseline and FEP 1 year.

The MMN peak amplitudes measured at the Fz and FCz electrode sites, which showed significant group differences between FEP patients and HCs, were analyzed to compare FEP baseline and FEP 1 year. **Figure 2A** compares the grand-averaged MMN of FEP baseline and FEP 1 year. **Figure 2B** shows the peak amplitudes at the Fz and FCz electrode sites among the FEP baseline and FEP 1 year. **Figure 2C** displays topographic maps of the MMN amplitudes of the FEP baseline and FEP 1 year. Paired t-tests indicated that the MMN amplitudes at Fz (p = 0.044) were significantly smaller in those with FEP at 1 year than at baseline. In terms of the MMN latency, the latency of FEP 1 year became longer than that of FEP baseline at Fz (p = 0.003) and FCz (p = 0.004; **Table 2**).

### Correlation of Longitudinal Change of MMN and of Clinical Status or Cognitive Functioning

**Figure 3:**
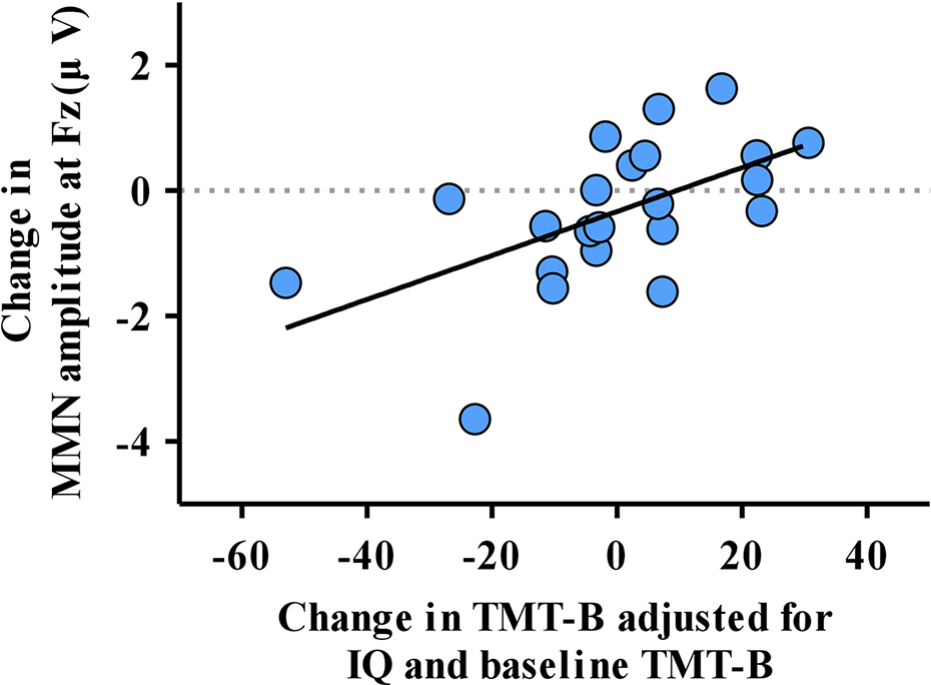
The correlation of the change in mismatch negativity (MMN) amplitude at Fz and change in the reaction time of the Trail Making Test part B (TMT-B) adjusted for significant factors obtained from multiple regression analysis.

The multiple regression analysis showed that the change in TMT-B correlated with the change in MMN amplitude at Fz (β = 9.057, 95% confidence interval [95% CI] = 1.426 to 16.688, p = 0.023), IQ (β = −0.683, 95% CI = −1.222 to −0.144, *p* = 0.016) and baseline TMT-B (β = 0.880, 95% CI = 0.708 to 1.051, p < 0.001; **Table 3**, **Figure 3**). However, the change in MMN amplitude at Fz did not show a correlation with the change in symptoms, functional outcome, TMT-A or TMT B/A during the 1-year follow-up period. The variance inflation factor (VIF) was less than 10 for all the significant predictors in the multiple regression analyses. In addition, the change in MMN latency at Fz or FCz did not correlate with the change in PANSS positive, negative, general, GAF, TMT-A or TMT-B in the multiple regression analysis (**Supplementary Table 3**).

**Table 3:** Significant factors correlated with changes in clinical symptoms, general function, and cognitive function.

Outcome variablesChange in	Significant factors	R^2^	Beta	P	95% CI	
					Lower	Upper
PANSS						
Total	Baseline PANSS total	0.808	0.846	<0.001^**^	0.653	1.040
	Antipsychotics		−0.467	0.002^*^	−0.750	−0.184
Positive symptoms	Baseline PANSS positive	0.830	0.893	<0.001^**^	0.713	1.074
	Antipsychotics		−0.071	0.042^*^	−0.139	−0.003
Negative symptoms	Sex	0.841	4.503	0.002^*^	1.830	7.175
	DUP		−0.669	0.010^*^	−1.156	−0.181
	Baseline PANSS negative		0.736	<0.001^**^	0.552	0.919
	Antipsychotics		−0.214	<0.001^**^	−0.314	−0.114
General symptoms	Baseline PANSS general	0.778	0.844	<0.001^**^	0.637	1.052
	Antipsychotics		−0.163	0.036^*^	−0.314	−0.012
GAF	Sex	0.597	−8.979	0.024^*^	−16.647	−1.312
	Baseline GAF score		0.464	<0.001^**^	0.247	0.680
TMT-A (n = 23)	Baseline TMT-A	0.448	0.698	<0.001^**^	0.365	1.031
TMT-B (n = 22)	Change in MMN amplitude at Fz	0.891	9.057	0.023^*^	1.426	16.688
	IQ		−0.683	0.016^*^	−1.222	−0.144
	Baseline TMT-B		0.880	<0.001^**^	0.708	1.051
TMT B/A (n = 22)	Baseline TMT B/A	0.855	0.992	<0.001^**^	0.801	1.182

PANSS, Positive and Negative Syndrome Scale; DUP, duration of untreated psychosis; GAF, Global Assessment of Functioning; TMT-A, Trail Making Test part A; TMT-B, Trail Making Test part B; TMT B/A, ratio score of Trail Making Test part B to part A; MMN, Mismatch negativity.

*The mean difference is significant at the 0.05 level.

**The mean difference is significant at the 0.005 level.

## Discussions

The aim of this study was to examine the longitudinal change in MMN during 1 year of usual treatment in patients with FEP. In line with previous studies (46–48), MMN amplitudes were significantly attenuated in FEP patients compared to HCs at baseline. MMN impairment at Fz worsened longitudinally although patients had significantly lower positive, negative, and general symptoms and showed better general functioning at follow-up. In addition, the reduction in MMN amplitude showed a significant correlation with the prolonged reaction time of TMT-B over 1 year. However, changes in MMN amplitude did not correlate with symptomatic or functional improvements in FEP patients.

These results indicate that MMN amplitude progressively became impaired over time regardless of symptomatic and functional improvement. The result of progressive worsening of MMN was consistent with previous results from Salisbury et al. (36) and Kaur et al. (34). These results might reflect the progressive pathophysiological changes of auditory cortex regions, which are known to generate MMN (49). In particular, the association between MMN progressive reduction and longitudinal decrease in left Heschl’s gyrus volume in FEP patients indicated that pathological alterations occur in the early stage of schizophrenia (36) although only a few reports have directly shown the relationship between MMN and structural change in schizophrenia (36, 50, 51). Since the average DOI of FEP patients in our study was 9.1 months, and SD was 6.9 months, longitudinal follow-up assessments were administered approximately 14 to 29 months after the onset of psychosis. The progression of MMN impairment during this period is consistent with the result of Erickson et al. (24), which suggested that the gradual progression of MMN impairment occurs within the first year or two after onset. Incompatibility with the result of Koshiyama et al. (37), which reported no progressive impairment when longitudinally comparing FEP, CHR, and HC groups, might have arisen from the varied follow-up durations among the groups and relatively small sample size in the study.

In the current study, a reduction in MMN amplitude was related to worsened performance in TMT-B over 1 year in FEP patients. TMT-B reflects higher-order cognitive functions, including working memory and/or cognitive flexibility and task-switching ability (52–54), and is not attributed to the localized activity of certain brain regions but derived from coordinated activities of brain networks (42). Therefore, the progressive MMN impairment associated with worsening performance in TMT-B found in this study may be reflective of progressive pathophysiological change during the early years after the onset in FEP patients.

On the other hand, MMN change was not significantly correlated with symptomatic or functional improvement over 1 year in FEP patients. This finding is in line with previous cross-sectional studies that reported MMN was not associated with clinical symptoms and GAF in FEP (55–57). In chronic schizophrenia patients, attenuation of MMN amplitude is stabilized and related to scores on the GAF (25, 27). Possibly, the correlation between GAF and MMN has been observed because cognitive deficits and functional impairment are stabilized in the chronic stage, and overall functioning is mainly influenced by cognitive deficits (58). However, in patients with FEP, in contrast to those with chronic schizophrenia, GAF is directly influenced by acute psychotic symptoms (59, 60) resulting in no association between GAF and MMN. According to our results, the MMN change itself might reflect changes in cognitive function at the early stages of psychosis such as in FEP.

In our results, the MMN latency of FEP patients became significantly longer after 1 year than at baseline. In contrast to MMN amplitude, MMN latency deficit has not been reported consistently, and its relationship with clinical outcome is still unclear in schizophrenia (61). Additionally, little is known about the longitudinal change in MMN latency. In this study, the change in PANSS positive, negative, general, GAF, TMT-A, and TMT-B did not correlate with the change in MMN latency at Fz or FCz in the multiple regression analysis. However, latency prolongation might reflect delayed auditory sensory discrimination demonstrating progressive brain pathology, although the result should be reproduced in other longitudinal studies to elucidate the clinical significance of this finding.

The diagnostic heterogeneity of FEP (62) results in a markedly diverse trajectory of the disorder. In our study, 16 patients showed progressive impairment of MMN amplitude at the Fz site, while 9 showed improvement (**Supplementary Figure 1**) although overall MMN progressed over time. This shows that the longitudinal course of MMN, which may reflect the underlying pathophysiological process, differs among patients with FEP. In this study, we showed that the change in MMN during the early phase of psychotic disorder was correlated with the change in cognitive functioning. This implies that if MMN impairment progresses during the follow-up, cognitive function tends to become impaired during the period. This suggests the possibility that patients with progressive impairment of MMN will have poorer prognosis than those without progression of MMN during the first episode of psychosis although larger studies are required to analyze different patient subgroups with different pathophysiological trajectories. Thus, this result may suggest the possibility of MMN being utilized as a potential biomarker for detecting cognitive prognostic trajectories of FEP patients. If MMN reflects the underlying pathophysiology of first-episode psychosis, one might consider the possibility that agents capable of altering MMN could decelerate pathophysiologic progression in the psychotic process (49). For example, previous studies have shown that N-acetyl-cysteine (NAC), a glutathione precursor, improved MMN (63), as well as clinical symptoms and cognitive impairments (64, 65) in schizophrenia patients. These results suggest that MMN may be used as a biomarker for testing and developing potential new therapeutic interventions that aim to decelerate pathophysiologic progression in the psychotic process.

There are several limitations in this study. First, among 36 FEP patients initially recruited at baseline, 25 patients participated in the 1-year follow-up ERP measurement. There is a possibility that patients who were lost to follow-up might have had different disease trajectories than those who completed the study, contributing to selection bias. Therefore, the association between MMN change and different prognostic trajectories across FEP patients could not be examined. Second, all FEP patients were taking medications, especially antipsychotics, which might have influenced the results of the MMN and the TMT. However, previous studies have shown that the usage of medications does not significantly affect MMN (57). In addition, the results from previous studies have shown that antipsychotics, especially second-generation antipsychotics, do not have negative impacts on TMT-A or -B performance (66, 67). Veselinovic et al. suggested that TMT-A performance was improved, and TMT-B performance was not changed during 6 months of second-generation antipsychotic treatment (66) although the long-term effects of antipsychotics on cognitive functions should be further studied. Third, longitudinal follow-up of MMN was performed only in FEP patients but not in HCs. Although one year would be relatively short to reflect age-related changes in MMN considering previous studies have demonstrated age-related effects using at least 10-year intervals (68, 69), the possibility that the change in MMN over a year in FEP patients might be due to age-related decline in MMN (70) should be considered when interpreting the results of the current study. Lastly, the follow-up duration of our study was one year in FEP, which made it difficult to elucidate the course of MMN and its correlation with clinical outcomes after 2 or 3 years of onset. Therefore, future longitudinal studies with larger sample sizes, longer durations of follow-up and inclusion of follow-up data for HCs are needed to complement the limitations of the current study.

## Conclusion

In conclusion, the present results suggest that progressive impairments in MMN amplitude and latency occur during the early years of psychotic disorder. In addition, this study is the first investigation to show that MMN amplitude reduction may be more closely related to underlying pathophysiological changes associated with cognitive change, rather than the symptomatic and functional improvements over several years after the onset of psychotic disorder. Therefore, MMN could be a potential biomarker not only for detecting heterogeneous prognostic trajectories of FEP patients but also for developing and testing new therapeutic interventions that aim to decelerate pathophysiologic progression in the psychotic process. Future studies are warranted to confirm the usefulness of MMN suggested by current study results and elucidate the relationship with the differential disease pathophysiology reflected by the heterogeneous prognostic trajectories of FEP and MMN.

## Data Availability Statement

The datasets generated for this study are available on request to the corresponding author.

## Ethics Statement

The studies involving human participants were reviewed and approved by Institutional Review Board of SNUH. Written informed consent to participate in this study was provided by the participants’ legal guardian/next of kin.

## Author Contributions

SL and MK designed the study. SL collected the data, performed statistical analysis and wrote the first manuscript. MK and JK interpreted the data and critically edited the manuscript. JP, WH, SO, and S-YM collected the data and revised the manuscript. MK and JK contributed to the conception of the study, interpreted the data, and gave critical comments on the manuscript. All authors contributed to the article and approved the submitted version.

## Funding

This work was supported by the Brain Research Program and the Basic Science Research Program through the National Research Foundation of Korea (NRF), funded by the Ministry of Science, ICT & Future Planning (grant nos. 2017M3C7A1029610 and 2019R1C1C1002457).

## Conflict of Interest

The authors declare that the research was conducted in the absence of any commercial or financial relationships that could be construed as a potential conflict of interest.
