# Supplementary material for: Progressive Impairment of Mismatch Negativity Is Reflective of Underlying Pathophysiological Changes in Patients With First-Episode Psychosis
Source: Front Psychiatry. 2020 Jun 18;11:587. doi: 10.3389/fpsyt.2020.00587 (PMC7314980; doi:10.3389/fpsyt.2020.00587)
Supplement: Supplementary file 1 [file DataSheet_1.docx]

Supplementary Material

There was no significant group difference in baseline demographic and clinical characteristics between the first-episode psychosis (FEP) follow-up group and the lost-to-follow-up group (Supplementary Table 1). Additionally, mismatch negativity (MMN) amplitude or latency between the FEP follow-up group and the lost-to-follow-up group showed no difference (Supplementary Table 2). The Mann-Whitney U test was used for continuous variables, and the linear-by-linear test was used for categorical variables.

The changes in the scores on the Positive and Negative Syndrome Scale (PANSS) positive, negative, general, Global Assessment of Functioning (GAF), Trail making test, type A (TMT-A) and Trail making test, type B (TMT-B) did not correlate with the change in MMN latency at Fz or FCz in the multiple regression analysis (Supplementary Table 3).

Sixteen FEP patients showed progressive impairment of MMN amplitude at the Fz site, while 9 showed improvement (Supplementary Figure 1).

**Supplementary Table 1.** Baseline characteristics of the first-episode psychosis (FEP) follow-up and FEP lost-to-follow-up groups

|  | FEP follow-up group (N=25) | | FEP lost-to-follow-up group (N=11) | | Statistical Analysis^a^ |
| --- | --- | --- | --- | --- | --- |
|  | Mean | SD | Mean | SD | P |
| Age (years) | 23.1 | 5.2 | 23.5 | 4.2 | 0.735 |
| Sex (Male/Female) | 10/15 | | 4/7 | | 0.839 |
| Handedness (Right/Left/Both) | 23/2/0 | | 9/9/1 | | 0.209 |
| IQ | 101.9 | 16.3 | 99.3 | 13.3 | 0.984 |
| Education (years) | 13.6 | 2.3 | 13.0 | 1.8 | 0.612 |
| DUP (months) | 3.0 | 2.9 | 4.2 | 3.9 | 0.456 |
| DOI (months) | 9.1 | 6.9 | 6.8 | 3.4 | 0.498 |
| PANSS |  |  |  |  |  |
| Total | 58.6 | 20.2 | 55.5 | 13.4 | 0.866 |
| Positive symptoms | 13.8 | 5.2 | 13.4 | 3.9 | 0.813 |
| Negative symptoms | 15.7 | 7.2 | 15.0 | 5.0 | 0.946 |
| General symptoms | 29.0 | 10.0 | 27.2 | 7.3 | 0.787 |
| TMT-A^b^ | 26.0 | 7.7 | 30.9 | 17.7 | 0.842 |
| TMT-B^b^ | 78.8 | 54.3 | 63.4 | 23.3 | 0.740 |
| TMT B/A^b^ | 3.01 | 1.50 | 2.33 | 0.94 | 0.170 |
| GAF | 54.5 | 17.7 | 51.7 | 7.9 | 0.359 |
| Antipsychotics dose^c^ | 17.2 | 13.2 | 13.2 | 9.3 | 0.636 |

IQ, Intelligent quotient; DUP, Duration of untreated psychosis; DOI, Duration of illness; PANSS, Positive and Negative Syndrome Scale; TMT-A, Trail Making Test part A; TMT-B, Trail Making Test part B; TMT B/A, ratio score of Trail Making Test part B to A; GAF, Global Assessment of Functioning.

^a^ derived from Mann-Whitney U test for continuous variables or linear-by-linear association test for categorical variables.

^b^ Missing values, n; TMT-A of FEP follow-up, 2; TMT-B of FEP follow-up, 3; TMT-A and B of FEP follow-up loss group, 3.

^c^ Mean olanzapine equivalent dose.

**Supplementary Table 2.** Mismatch negativity (MMN) amplitudes and latencies of the first-episode psychosis (FEP) follow-up and FEP lost-to-follow-up groups

|  | FEP follow-up group (N=25) | | FEP lost-to-follow-up group (N=11) | | Statistical Analysis^a^ |
| --- | --- | --- | --- | --- | --- |
|  | Mean | SD | Mean | SD | P |
| Amplitude (μV) |  |  |  |  |  |
| Fz | -1.8 | 1.0 | -1.6 | 0.9 | 0.685 |
| FCz | -1.7 | 1.3 | -1.4 | 0.8 | 0.542 |
| Latency (ms) |  |  |  |  |  |
| Fz | 181.4 | 23.4 | 181.2 | 28.3 | 0.276 |
| FCz | 186.6 | 17.5 | 168.0 | 20.6 | 0.161 |
| ^a^ derived from Mann-Whitney U test | | | | | |

**Supplementary Table 3.** Significant factors correlate with change in clinical symptoms, general functioning and cognitive function

| Outcome variables |  |  |  |  | 95% CI | |
| --- | --- | --- | --- | --- | --- | --- |
| Change in | Significant Factors | R^2^ | Beta | P | Lower | Upper |
| PANSS |  |  |  |  |  |  |
| Total | Antipsychotics | 0.774 | -0.431 | 0.006^*^ | -0.728 | -0.135 |
|  | Baseline PANSS total |  | 0.852 | <0.001^**^ | 0.648 | 1.057 |
| Positive | Antipsychotics | 0.830 | -0.071 | 0.042^*^ | -0.139 | -0.003 |
|  | Baseline PANSS positive |  | 0.893 | <0.001^**^ | 0.713 | 1.074 |
| Negative | Sex | 0.836 | 4.406 | 0.003^*^ | 1.697 | 7.115 |
|  | DUP |  | -0.739 | 0.006^*^ | -1.243 | -0.236 |
|  | Antipsychotics |  | -0.217 | <0.001^**^ | -0.318 | -0.115 |
|  | Baseline PANSS negative |  | 0.731 | <0.001^**^ | 0.545 | 0.917 |
| General | Baseline PANSS general | 0.734 | 0.831 | <0.001^**^ | 0.610 | 1.052 |
| GAF | Sex | 0.597 | -8.979 | 0.024 | -16.647 | -1.312 |
|  | IQ |  | 0.212 | 0.076 | -0.024 | 0.449 |
|  | Baseline GAF |  | 0.464 | <0.001^**^ | 0.247 | 0.680 |
| TMT-A (n=23) | Baseline TMT-A | 0.447 | 0.697 | <0.001^**^ | 0.345 | 1.048 |
| TMT-B (n=22) | Baseline TMT-B | 0.851 | -0.575 | <0.001^**^ | 0.666 | 1.053 |
| TMT B/A (n=22) | Baseline TMT B/A | 0.855 | 0.992 | <0.001^**^ | 0.801 | 1.182 |

PANSS, Positive and Negative Syndrome Scale; DUP, Duration of untreated psychosis; GAF, Global Assessment of Functioning; IQ, Intelligence Quotient; TMT-A, Trail Making Test part A; TMT-B, Trail Making Test part B; TMT B/A, ratio score of Trail Making Test part B to A.

^*^ The mean difference is significant at the 0.05 level.

^**^ The mean difference is significant at the 0.005 level.


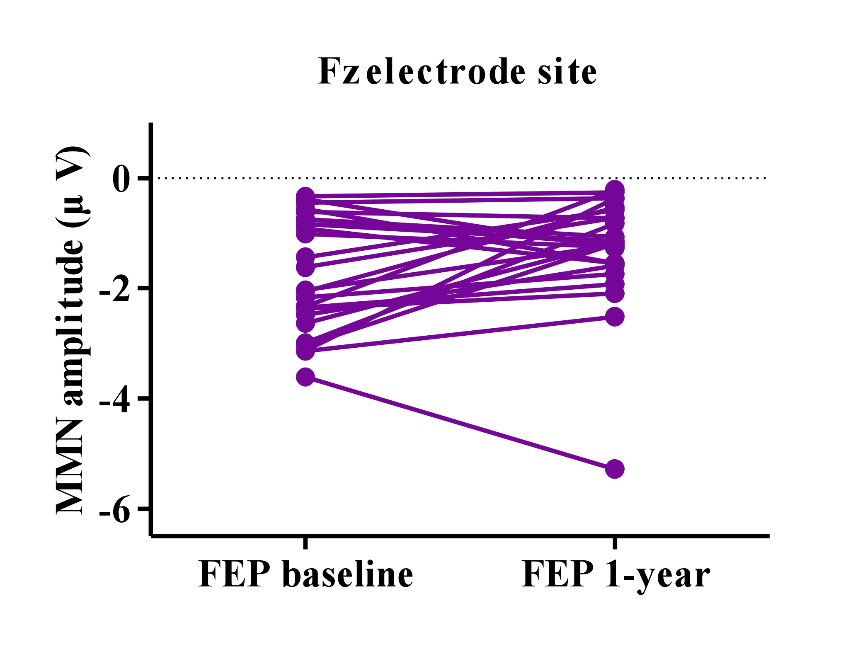


**Supplementary Figure 1.** Longitudinal change in mismatch negativity (MMN) amplitude at the Fz site in individuals. Each line indicates the longitudinal change in MMN amplitude in each FEP patient.
